# A randomized prospective mechanistic cardiac magnetic resonance study correlating catheter stability, late gadolinium enhancement and 3 year clinical outcomes in robotically assisted vs. standard catheter ablation

**DOI:** 10.1093/europace/euu364

**Published:** 2015-02-16

**Authors:** Aruna Arujuna, Rashed Karim, Niloufar Zarinabad, Jaspal Gill, Kawal Rhode, Tobias Schaeffter, Matthew Wright, C. Aldo Rinaldi, Michael Cooklin, Reza Razavi, Mark D. O'Neill, Jaswinder S. Gill

**Affiliations:** 1 Division of Imaging Sciences and Biomedical Engineering, King's College London, St. Thomas′ Hospital, London SE1 7EH, UK; 2 Department of Cardiology, Guy's and St. Thomas’ NHS Foundation Trust, London, UK

**Keywords:** Atrial fibrillation, Robotic-assisted, Catheter ablation, Pulmonary vein isolation, Cardiac magnetic resonance imaging, Long-term follow-up

## Abstract

**Aims:**

To prospectively compare cardiac magnetic resonance late gadolinium enhancement (LGE) findings created by standard vs. robotically assisted catheter ablation lesions and correlate these with clinical outcomes.

**Methods and results:**

Forty paroxysmal atrial fibrillation patients (mean age 54 ± 13.8 years) undergoing first left atrial ablation were randomized to either robotic-assisted navigation (Hansen Sensei^®^ X) or standard navigation. Pre-procedural, acute (24 h post-procedure) and late (beyond 3 months) scans were performed with LGE and T2W imaging sequences and percentage circumferential enhancement around the pulmonary vein (PV) antra were quantified. Baseline pre-procedural enhancements were similar in both groups. On acute imaging, mean % encirclements by LGE and T2W signal were 72% and 80% in the robotic group vs. 60% (*P* = 0.002) and 76%(*P* = 0.45) for standard ablation. On late imaging, the T2W signal resolved to baseline in both groups. Late gadolinium enhancement remained the predominant signal with 56% encirclement in the robotic group vs. 45% in the standard group (*P* = 0.04). At 6 months follow-up, arrhythmia-free patients had an almost similar mean LGE encirclement (robotic 64%, standard 60%, *P* = 0.45) but in recurrences, LGE was higher in the robotic group (43% vs. 30%, *P* = 0.001). At mean 3 years follow-up, 1.3 procedures were performed in the robotic group compared with 1.9 (*P* < 0.001) in the standard to achieve a success rate of 80% vs. 75%.

**Conclusion:**

Robotically assisted ablation results in greater LGE around the PV antrum. Effective lesions created through improved catheter stability and contact force during initial treatment may have a role in reducing subsequent re-do procedures.

**What's new?**

- This is the first cardiac magnetic resonance (CMR) study performed to prospectively compare the effect of robotic and standard catheter ablation by late gadolinium enhancement and evaluate the findings in relation to 6 month and 3 year clinical outcome.
- Robotic-assisted ablation results in greater percentages of permanent pulmonary vein (PV) encirclement quantified on CMR, suggesting better catheter contact and stability.
- A significantly less LGE signal regression from acute to late scan in the robotic recurrences group suggests that acute energy delivery with this approach produces more durable lesions.
- A significantly lower number of re-do procedures was observed in the robotic group over the mean 3 years clinical follow-up period.
- These data represent the first CMR evidence in man that robotic-assisted ablations create more efficacious ablation lesions than a standard manual approach.

## Introduction

Arrhythmia recurrences following pulmonary vein isolation (PVI) in paroxysmal atrial fibrillation (PAF) are almost universally associated with electrical reconnection between the left atrium (LA) and pulmonary veins (PVs).^1^ Acute PV electrical isolation achieved following energy delivery to the left atrial-pulmonary vein (LA-PV) junction or antrum^2^ does not always translate into long-term clinical success, with only 50–60% of patients being cured following a single procedure.^3,4^ The formation of a durable permanent transmural scar is critical to block electrical conduction between the LA and PVs and to prevent spontaneous PV ectopics from triggering AF.

Over the last 5 years, studies have confirmed that radiofrequency (RF) lesions within the LA can be visualized using late gadolinium enhancement (LGE) cardiac magnetic resonance (CMR) imaging.^5–9^ A good correlation between greater amounts of LGE quantified following ablation and a successful clinical outcome has been demonstrated.^7^ In the acute setting, T2-weighted imaging enables edema visualization while LGE imaging is likely to represent both necrotic and inflamed tissue.^10^ In the chronic scans, it is possible that areas of non-necrotic tissue may either recover or progress to form a scar; the latter case is distinguished by LGE imaging. The more complete the encirclement by tissue necrosis around the PV, the less likely that LA to PV conduction will be restored.

It has been shown that RF lesion depth and dimensions are heavily influenced by contact force,^11^ catheter tip orientation and catheter stability.^12^ The use of steerable introducers, high-frequency jet ventilation, and robotic assistance has been shown to improve catheter stability. The Hansen-Robotic-System has an incorporated pressure sensing mechanism (IntelliSense) to determine the force exerted and the ability to maintain a stable catheter tip position.^13^ To date, the correlations between catheter stability, improved delivery of RF energy and enhanced occurrence of irreversible tissue injury have been assessed by clinical outcomes and freedom from arrhythmia.

This mechanistic study is the first to examine the role of catheter stability during the index PVI procedure and correlate this with tissue injury via CMR late gadolinium findings.

## Methods

### Patient population

Forty patients (28 male, mean age 54 ± 13.8 years) with symptomatic, drug refractory PAF undergoing their first PVI were randomly assigned to robotically assisted vs. standard wide area circumferential ablation. Randomization was performed based on the procedural admission day. Robotic-assisted catheter ablation was performed on all PAF patients admitted on the second day of the week while patients attending on the first day and fourth day of the week underwent a standard catheter ablation. All scans used for the purposes of data analysis were deemed of adequate quality for analysis by an experienced CMR operator. Therapeutic anti-coagulation with an INR >2 for at least 4 weeks prior to the procedure was mandated. The study was approved by the local research ethics committee.

Acute procedural success was defined as isolation of all PVs, confirmed using a circumferential mapping catheter. Patients were followed in clinic to assess symptoms. Twenty-four hour Holter monitors were performed at 6 months from the index procedure. Every effort was made to obtain electrocardiogram (ECG) recordings of symptomatic recurrences. Recurrences were defined on the basis of (i) symptoms with ECG evidence of the presence of any sustained atrial arrhythmia or (ii) the presence of symptomatic or asymptomatic episodes of atrial arrhythmia lasting for >30 s on ambulatory cardiac monitoring. All patients with recurrences were offered re-do procedures. The use of subsequent anti-arrhythmics following ablation was determined on clinical needs. The total number of re-do ablations and subsequent outcomes over 3 years are presented.

### Magnetic resonance image acquisition

The CMR sequences used in a 1.5 Tesla Philips Achieva MR system (Philips Healthcare) to acquire the images have been described previously.^5^ In brief, T2-weighted images were acquired via a multi-slice turbo spin echo technique with a double inversion recovery pre-pulse for black-blood imaging. Late gadolinium enhancement visualization was obtained via a 3D ECG-triggered, free-breathing inversion recovery turbo field echo scan, 20 min following contrast agent administration. Images were acquired at three time points: (1) prior to ablation, (2) within 24 h of ablation; and (3) 3–6 months following ablation.

### Ablation settings

A three-dimensional (3-D) geometry of the LA was created using either NavX^™^ (St. Jude Medical Inc.) or CARTO XP (Biosense Webster Inc.). Wide area circumferential ablation was performed in all the patients. In the robotically navigated group, 30 s energy delivery with power settings of 25 W on the anterior wall and 20 W on the posterior wall with target temperatures of 40–42°C were delivered. If LA-PV conduction persisted despite wide area circumferential ablation, additional lesions were delivered at the sites of earliest activation on the circular mapping catheter until entry block in all four veins was confirmed by observing the elimination or dissociation of PV potentials. For standard ablation, power was 30 W on the anterior wall and 25 W on the posterior wall, limited to up to at 60 s energy delivery.

### Image processing and analysis

T2W and LGE signal circumferential quantification were performed by reconstructing all CMR scans into individual left atrial shells using a semi-automated 3-D visualization method.^9^ Areas of LGE or high T2W signal intensity were defined as being more than three standard deviations above the mean signal intensity of the simultaneously imaged ventricular myocardium as previously valated.^9,14^ The LA surface was colour coded according to the maximum-intensity projection values, ranging from green (minimum) to red (maximum).

For each PV pair, T2W and LGE were quantified as occupying a percentage of the antral circumference. The % circumferential LGE, T2-weighted signal and combination of LGE and T2 (LGE+T2) encircling the PVs were quantified. All 3D MR reconstructions were analysed twice independently by two experienced readers, blinded to clinical outcome and to the timing of the scan following catheter ablation. A high degree of inter-observer agreement was seen on a Bland Altman test with a maximum observed difference of 10% . The mean ± SD inter-observer error for LGE, T2W, and LGE&T2 was 1.5 ± 2.5%, 1.5 ± 3.5%, and 1.0 ± 2.2% which was acceptable for the purposes of data analysis.

### Statistical analysis

Summaries for continuous variables are expressed as mean ± SD. Follow-up times are reported as median and interquartile range (25th, 75th percentile). Categorical variables were compared among recurrences and non-recurrences groups using a χ^2^ test. The % circumferential encirclement by LGE, T2, and LGE&T2 groups were compared with test for differences between group means. Statistical analyses were performed using Stata (StataCorp 2011) and Matlab (The Mathworks, 2012b). Analysis of variance was used to compare robotic-assisted outcome with standard group results. A *P* value of < 0.05 was considered statistically significant. Event-free survival curves were estimated by the Kaplan–Meier product-limit method.

## Results

### Patient and procedural data

**Figure 1:**
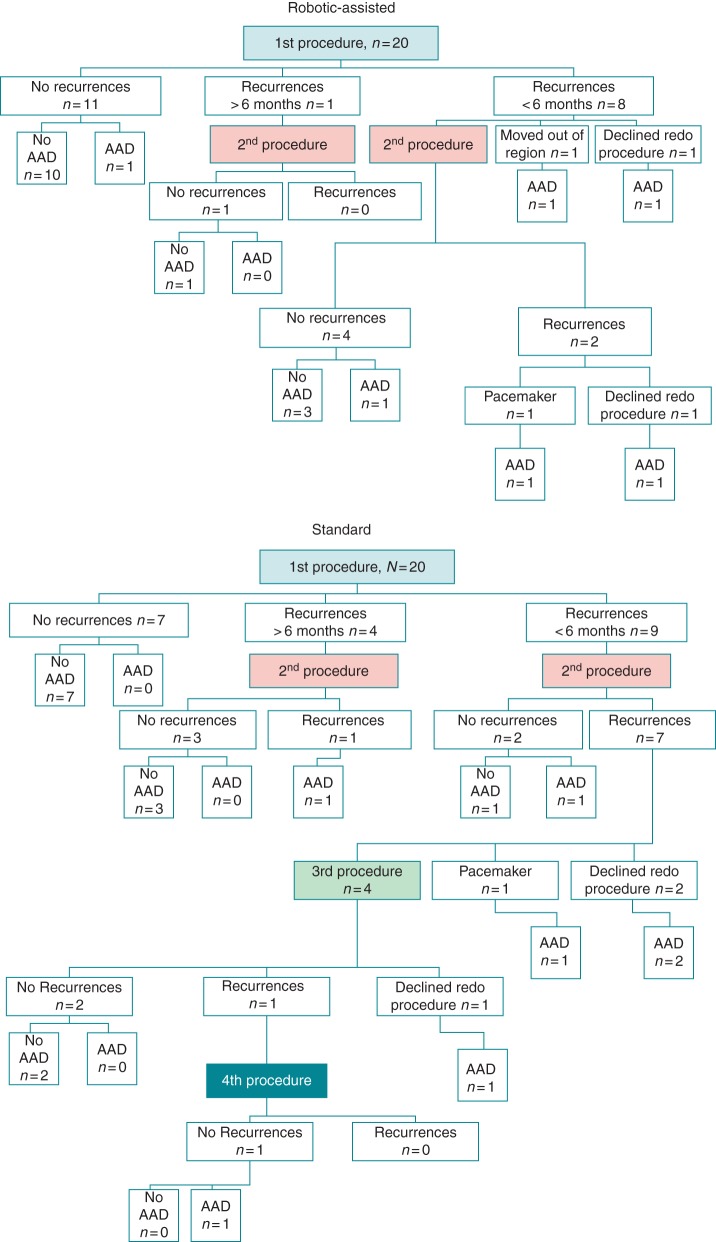
Flow chart outlining number of times procedure performed, outcome at each stage and the use of anti-arrhythmic drugs. Both groups had 20 patients each undergoing first ablation procedure. In the robotic group, seven patients underwent a second procedure while three patients did not undergo repeat procedures and one patient required a pacemaker. In the standard group, 13 patients underwent a second procedure, four patients a third procedure, and one patient undergoing a fourth procedure. Three patients declined further procedures and one patient underwent a pacemaker implantation.

**Figure 2:**
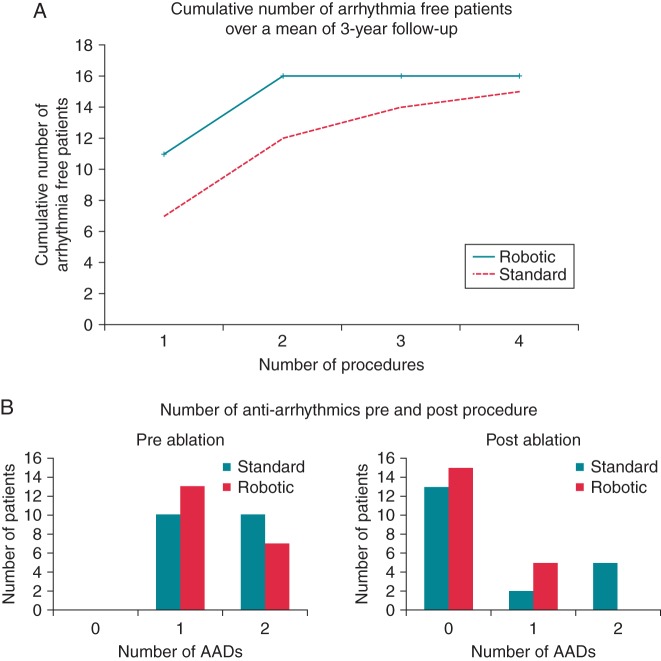
(*A*) Cumulative number of arrhythmia free patients over a 3 years mean follow-up period. (*B*) Anti-arrhythmic use pre- and post-ablation in both groups at follow-up.

*Table 1* outlines the clinical characteristics of the study population in both groups. Successful PV isolation was achieved in all patients without a significant difference in RF energy delivered between groups. At 6 month follow-up, 12 patients (60%) in the robotic group vs. 11 patients (55%) in the standard group were arrhythmia free. Median time to recurrence was 78 days (32–146 days) for the robotic group vs. 96 days (68–167 days) in the standard group. At median 3-year follow-up (2.2–3.5 years) (*Figure 1*), 16 patients (80%) in the robotic group vs. 15 patients (75%) in the standard group achieved freedom from arrhythmia following 26 vs. 38 procedures (*P* = 0.0008). Patients in the robotically assisted group experienced an average of 1.3 in comparison to 1.9 procedures per patient in the standard group. Seven patients with recurrences underwent one re-do procedure in the robotically assisted group. In contrast, patients in the standard group undergoing two procedures were 13, undergoing three procedures were 4, and undergoing four procedures one. Median time interval between index and first re-do procedure was 30 (21–41) weeks in the robotic group vs. 55 (43–88) weeks in the standard (*P* = 0.3). One patient in each group received a pacemaker (*Figure 1*). The maximum cumulative number of arrhythmia-free patients is achieved faster in the robotically assisted group (*Figure 2A*).

**Table 1:** Baseline characteristics

	Total population (*N* = 40)	Robotic navigation (*N* = 20)	Manual catheter ablation (*N* = 20)	*P* value
Male, *n* (%)	28 (70)	15 (75)	13 (65)	0.53
Age (years)	54 ± 13.8	54 ± 14.3	55 ± 11.2	0.86
AF duration (months)	30 ± 11.2	32 ± 9.1	30 ± 12.6	0.78
LA size (cm)	3.4 ± 3.5	3.4 ± 0.3	3.5 ± 0.2	0.92
LVEF, %	56 ± 8	58 ± 6	56 ± 8	0.86
Hypertension	11	7	4	>0.10
Diabetes	1	0	1	>0.10
Coronary artery disease	1	1	0	>0.10
Thyroid	2	2	0	>0.10
Smoking	6	4	2	>0.10
Fluoroscopy dose (Gycm^2^)	1227 ± 667	1291 ± 607	1141 ± 755	0.53
Procedure time (min)	210 ± 26.1	220 ± 21.4	199 ± 27.7	0.06
Number of applications	134 ± 42	141 ± 33	126 ± 58	0.63
Energy delivered (Joules)	84201 ± 28855	88391 ± 28519	80011 ± 28338	0.46

With regard to anti-arrhythmic use at the time of the procedure, 10 patients in the standard group were on two agents and 10 were on a single agent. Following catheter ablation, at mean 3 years follow-up, 13 patients were on no anti-arrhythmic drugs (AAD's), 2 were on one agent, and five patients were on two AAD's (*Figure 2B*). Conversely in the robotic group, 13 patients were on one agent and 7 patients on two agents pre-procedure. Following catheter ablation at a mean 3 years follow-up, only six patients were on a single AAD (*Figure 2B*).

Procedural complications following the index procedure included two femoral venous haematomas, resolved with conservative management and one pseudo-aneurysm not requiring surgical intervention. No stroke, tamponade, or oesophageal fistula occurred in this study and no PV stenosis was detected on follow-up magnetic resonance imaging.

### Cardiac magnetic resonance evaluation within 24 h post catheter ablation (acute scans) and 3–6 months post catheter ablation (follow-up scans)

**Figure 3:**
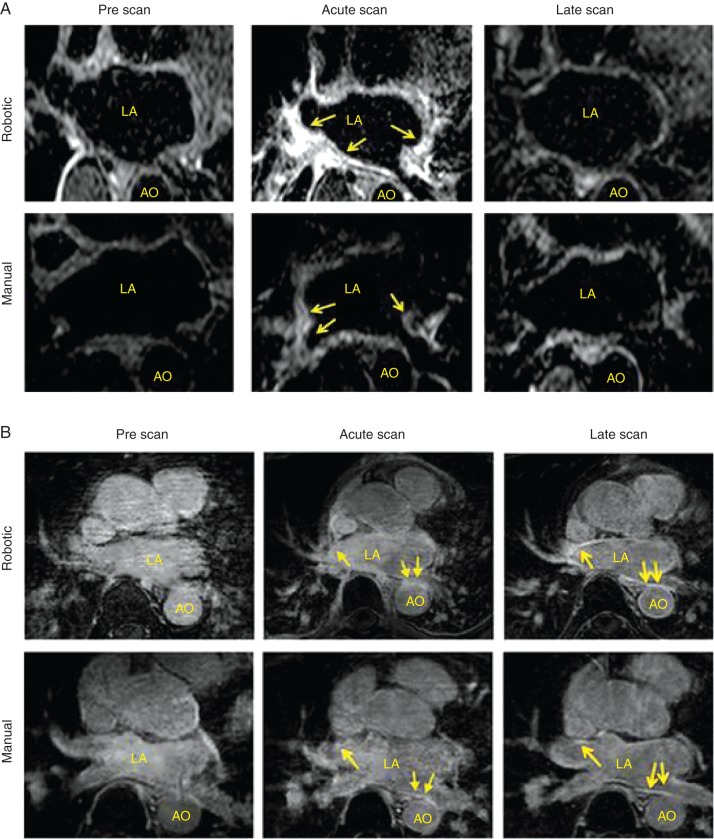
Serial T2W (*A*) and LGE (*B*) CMR scans performed on a single patient following robotic and standard navigated catheter ablation at 3 time points. In the T2W series, both groups demonstrate an increase in signal intensity and atrial wall thickness following ablation which resolves on the late follow-up scans. In the LGE series (*B*), baseline images in the first column show no significant DE signal (tissue injury/necrosis) compared with acute post-ablation images in the second column. The late scans in the third column shows that areas of LGE signal become less diffuse and more defined with sharper borders in comparison to the acute scans.

**Figure 4:**
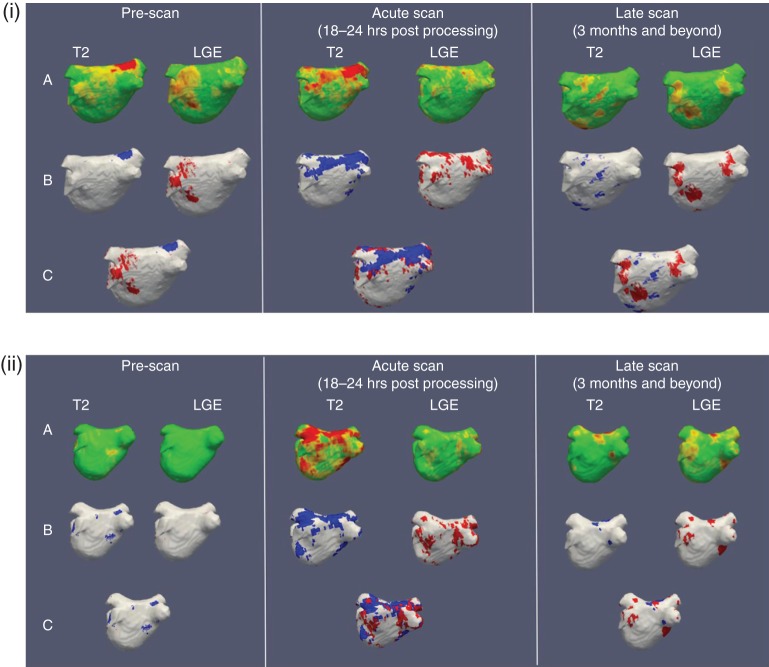
Example of a series of 3-D LA reconstructed shells (at 3 time points) in two patients to compare robotic (*A*) vs. standard catheter ablation (*B*). Blue represents areas of T2 signal while red represents LGE. More ‘islands' of red are seen in the robotic assisted LA shell implying greater tissue injury achieved. The first row represents the raw data transferred onto the atrial shell, each for T2 and LGE (Row A). The second row represents the semi-automated quantification of T2 and LGE signals (Row B). The third row demonstrates the combination of T2 and LGE from the second row onto a single atrial shell (Row C).

Baseline circumferential burdens of LGE and T2-weighted signal prior to any ablation were similar in both groups and did not occupy more than 5% of the PV circumference. Post-ablation acute imaging was performed between 18 and 24 h following catheter ablation. *Figure 3* demonstrates the typical T2-weighted (*Figure 3A*) and LGE (*Figure 3B*) appearances in two patients before and after catheter ablation. The left atrial burdens of LGE and the T2-weighted signal were significantly increased following both catheter ablation approaches in comparison with pre-ablation. In general, the LGE signal was concentrated in the PV antral region while the T2W signal was more widely distributed in the atrium, remote from the sites of ablation. Combined analyses of the LGE and T2W signals using reconstructed shells co-displaying both signal types revealed areas of T2W enhancement overlapping and inter-digitating with the areas of high LGE signal intensity (*Figure 4*). Hence the total combined LGE and T2W percentage PV encirclement on the combined overlay shells was 100% or less.

On the early scans, mean circumferential extent obtained by a combination of LGE&T2 signal overlay was 94 ± 6% in the robotic group vs. 88% ± 10% (*P* = 0.03) in the standard group. Mean LGE encirclement was 72% ± 8% in the robotically assisted group vs. 60% ± 17% (*P* = 0.002) in the standard group. T2W signals, on the other hand, were relatively similar in both groups: 80% ± 12% and 76% ± 15% (*P* = 0.45) for robotically assisted vs. Standard, respectively.

**Figure 5:**
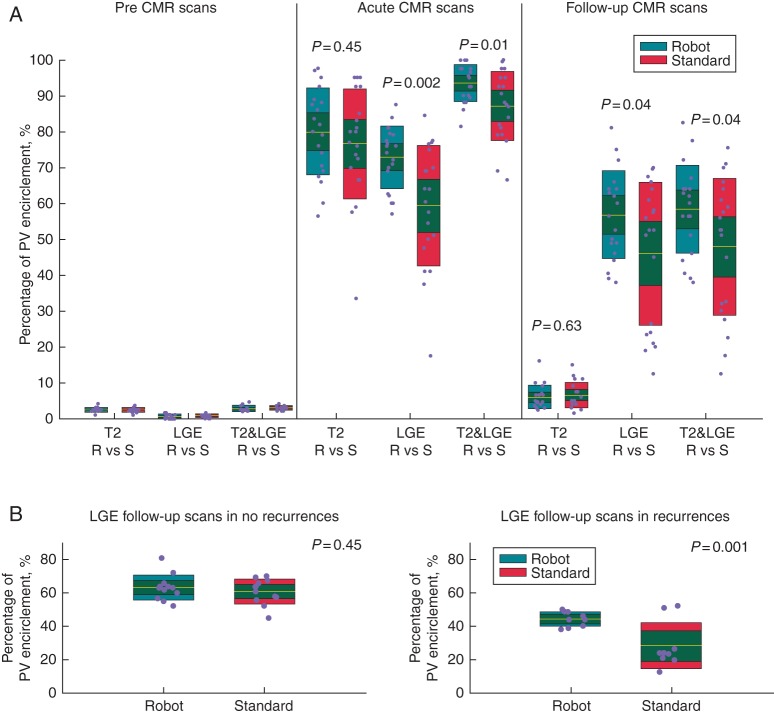
(*A*) Scatter-boxplot shows a comparison of pre, acute and late T2, LGE and combined T2&LGE for both robotic (green) and standard (brown) ablation. Each individual scatter plot represents the raw data for that specific group. The dots within each group have been dispersed horizontally to optimize visualization and clarity. The boxplots on the other hand represent median (red line), 95% confidence intervals (pink box) and 1 standard deviation (green/brown box). An overall higher enhancement is seen post-procedure in the robotic group compared with the standard group**.** The higher percentage encirclements by LGE assessment in the robotic group continued to remain significantly more in the follow-up scans. The overall lower standard deviation in the robotic group suggests better consistency in creating tissue injury. This is most likely a function of catheter stability resulting in better tissue contact. (*B*) Overall, a higher percentage encirclement is noted in the robotic group with statistical significance achieved in the recurrences cohort. This may have potential consequences for re-do ablation procedures.

On the follow-up scans the T2W signal had largely resolved (*Figure 5A*), while a decline in the extent of the LGE signal was seen in both groups. In the robotic group, the mean circumferential extent obtained by the LGE signal alone was 56 ± 11% in comparison to 45 ± 20% (*P* = 0.04) in the standard group. Overall, a higher mean percentage encirclement was consistently noted in the robotic group with an average 10% higher mean margin of encirclement observed on the chronic scans by LGE.

### Correlation between clinical outcome and late gadolinium enhancement assessment

In both robotic and standard groups, acute and follow-up scan data were analysed into two groups according to the respective clinical outcome—those with and without arrhythmia recurrences (*Figure 5B*).

In the robotic group, mean LGE percentage encirclements observed between no recurrences and recurrences were, 75 ± 8% vs. 66 ± 7% (*P* = 0.01) on the acute scans and 64 ± 8% vs. 43 ± 5%, (*P* < 0.0001) on the follow-up scans.

In the standard group, the corresponding LGE encirclement are 70 ± 9% vs. 45 ± 13% (no recurrences vs. recurrences, *P* = 0.0001) on acute imaging and 60 ± 8% vs. 30 ± 14% (no recurrences vs. recurrences, *P* < 0.0001) on follow-up imaging.

At the 6-month time point, more of the percentage encirclement observed around the PVs in the acute scans persisted through to the follow-up scans in the overall arrhythmia-free patient group (robotic vs. standard: acute scans −75 ± 8% vs. 70 ± 9%, *P* = 0.14 and follow-up scans 64 ± 8% vs. 60 ± 8%, *P* = 0.45). In the recurrences group, LGE was higher in the robotic group (acute scans −66 ± 7% vs. 45 ± 13%, *P* = 0.007 and follow-up scans 43 ± 5% vs. 30 ± 14%, *P* = 0.001). A higher co-efficient of variation is observed in the standard recurrences group 0.28 and 0.46 in comparison to the 0.1 and 0.11 in the robotic recurrences group (*Table 2*).

**Table 2:** Coefficient of variation between robotically assisted and standard ablation in acute and follow-up scans for no recurrences vs. recurrences

	Acute		Follow up	
	Robotically assisted	Standard	Robotically assisted	Standard
No recurrences	0.10	0.12	0.12	0.13
Recurrences	0.10	0.28	0.11	0.46

**Figure 6:**
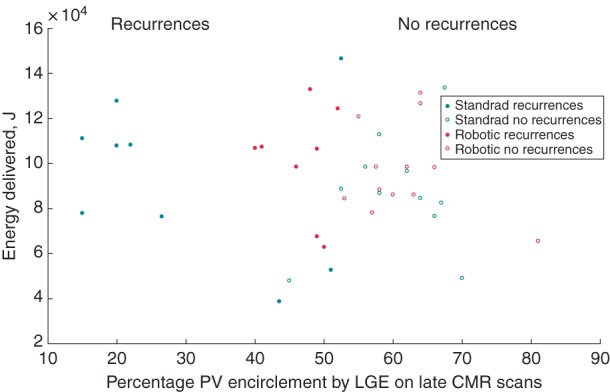
Scatter plot describes the relationship between energy delivered and scar created as quantified on LGE–CMR by percentage PV encirclement. Both robotic and standard ablation datasets have been categorized into no recurrences vs. recurrences. The blue circles representing robotic procedures appear less dispersed and have a minimum LGE value of around 40%. The red circles representing standard catheter ablation are widely dispersed and have a minimum LGE value of 15% to 30% despite high total energy delivery. This suggests better catheter–tissue contact conferred by the robotic system yielding higher percentages of PV antrum scar.

A comparison of energy delivered, follow-up CMR LGE and 6 months clinical outcome between the two groups is presented in *Figure 6*. In the standard group, there is no correlation between energy delivery and observed LGE on the follow-up scans. Even at high-energy delivery, low LGE values are observed. Conversely, the points on the robotic scatter plot are less spread and more close together. The % PV encirclement by LGE values are generally above 40% in robotically assisted procedures.

## Discussion

This is the first CMR study performed to prospectively compare the effect of robotic and standard catheter ablation by LGE and evaluate the findings in relation to 6 months and 3 years clinical outcome. The main findings of this study are (1) robotic-assisted ablation results in greater percentages of permanent PV encirclement quantified on CMR; (2) there was significantly less LGE signal regression from acute to late scan in the robotic recurrences group, suggesting that acute energy delivery with this approach produces more durable lesions; (3)at a set range of energy delivery, more robotic points with higher LGE was observed in the scatter plot, suggesting increased catheter contact and stability; (4) and significantly lower number of re-do procedures were observed over the mean 3 years clinical follow-up period. These data represent the first CMR evidence in man that robotic-assisted ablations create more efficacious ablation lesions than a standard manual approach.

Recently, improved electrogram signal attenuation during catheter ablation has been demonstrated in robotic navigated procedures.^15,16^ Both catheter stability and constant energy delivery were deemed key in achieving bipolar electrogram attenuation. This CMR study demonstrating increased LGE in robotic-assisted procedures provides a mechanistic explanation suggesting greater tissue injury as a result of greater catheter–tissue contact, leading to better electrogram attenuation. An approximate 50% electrogram attenuation achieved within 30 s of ablation in the standard group in comparison to 15 s in the robotic group was reported.

The findings of the Efficas studies strongly suggest that initial catheter contact force at the onset of RF delivery is a critical determinant of lesion quality, as assessed by the force time integral and lesion continuity index, with clinical outcome being predicted by the poorest quality lesion delivery and greater catheter instability.^17^ This study supports this hypothesis by demonstrating a 10% higher percentage encirclement observed on the robotic navigation system (RNS) late scans in comparison with the standard group. A lower regression of the LGE signal between the acute and late scans in the robotic recurrences group suggests the creation of a more durable lesion. The narrower range in % encirclements in the robotic group infers less variability in lesion delivery, suggesting better tissue contact. While this did not translate into improved clinical outcome after a single procedure, a reduction in the number of re-do procedures and anti-arrhythmic agent use is observed. It is important to note that acute LGE imaging is less likely to represent true scar as there is an overlap of injured tissue as well as inflammation. Late gadolinium enhancement quantified on late scans represents a better marker for true scar as the inflammation settles and injured tissue become more defined.

The association between energy delivered and scar formation analysed on the 6-month LGE CMR scans was examined. At a set range of energy delivery, more points with higher DE were observed on the robotic scatter plot. We hypothesise that this is attributable to better catheter control and stability conferred by the RNS. The better consistency and higher reproducibility inferred from both the smaller standard deviation and the more closely placed points on the scatter plot imply a relatively a higher precision in lesion delivery in the robotic group in comparison to the standard.

The 3 years clinical outcome findings suggest that the overall higher LGE observed in the robotically assisted group translates into a significantly less number of re-do procedures. These findings corroborate a similar mid-term study outcome previously reported.^18^ Durable tissue injury created by both better catheter–tissue contact and catheter stability reduces the need for multiple re-do ablation procedures. The implications here on overall patient wellbeing and health cost-efficiency warrant a further larger study. The overall greater reduction in the number of anti-arrhythmics pre-and post-ablation in the robotically assisted group suggests a better modification of the atrial tissue, resulting in better symptom control even in patients with recurrences.

## Study limitations

This is a small mechanistic study aimed at assessing the correlations between catheter stability, tissue injury, and clinical outcome. The true clinical impact of RNS-assisted catheter ablation on mid- to long-term clinical outcome would require a larger randomized study for validation of the findings. However, this was not the primary objective of this hypothesis-driven CMR assessment of atrial injury. Failure to detect asymptomatic recurrences of AF means that the incidence of asymptomatic AF is likely to be underreported in this study.

The correlation between contact-force and LGE lesion characteristics is not assessed here. A separate study evaluating this is planned.

## Conclusion

The increased LGE seen on CMR scans following robotic-assisted catheter ablation suggests a greater extent of tissue injury around the PV antrum in comparison with the standard approach. This is likely to be a function of better catheter stability and more effective lesion delivery.

## Funding

This work was supported in part by the Technology Strategy Board under Grant 17352, in part by the Philips Healthcare, Best, The Netherlands, and in part by the EPSRC under Grant EP/D061474/1. Dr Arujuna was supported by St Jude Medical. The Centre of Excellence in Medical Engineering is funded by the Wellcome Trust and EPSRC under grant number WT 088641/Z/09/Z. The authors also acknowledge financial support from the Department of Health via the National Institute for Health Research (NIHR) comprehensive Biomedical Research Centre award to Guy's & St Thomas' NHS Foundation Trust in partnership with King's College London and King's College Hospital NHS Foundation Trust. Funding to pay the Open Access publication charges for this article was provided by Wellcome Trust.
